# Investigating Asian American Adolescents’ Resiliency Factors and Young Adult Mental Health Outcomes at 14-year Follow-up: A Nationally Representative Prospective Cohort Study

**DOI:** 10.1007/s10903-022-01373-1

**Published:** 2022-07-11

**Authors:** Puja Iyer, Deepika Parmar, Kyle T. Ganson, Jennifer Tabler, Samira Soleimanpour, Jason M. Nagata

**Affiliations:** 1 Department of Pediatrics, University of California, San Francisco, Box 0110, 550 16th St, 4th Floor, 94143 San Francisco, CA USA; 2 Factor-Inwentash Faculty of Social Work, University of Toronto, Toronto, ON Canada; 3 Department of Criminal Justice and Sociology, University of Wyoming, Laramie, WY USA; 4 Institute for Health Policy Studies, University of California, San Francisco, CA San Francisco, USA

**Keywords:** Asian American, Adolescent, Mental Health, Resilience, Resiliency, Youth

## Abstract

There is scant research on how Asian American adolescents’ resiliency relates to mental well-being in adulthood. The objective of this study was to determine the prospective associations between resiliency factors (individual, family, and school community) in adolescence and mental health outcomes in adulthood, among a national sample of Asian Americans. We analyzed data from 1020 Asian American adolescents who were followed for 14 years in the National Longitudinal Study of Adolescent to Adult Health. Of the resiliency factors, individual self-esteem (Adjusted Odds Ratio [AOR] 0.54, 95% Confidence Interval [CI] 0.37–0.79) and family connectedness (AOR 0.78, 95% CI 0.65–0.93) in adolescence were found to be protective against adult mental health outcomes in logistic regression models adjusting for sociodemographic factors and baseline mental health. Our study identified individual and family resiliency factors which can be leveraged to help Asian American adolescents and families in cultivating better mental health.

## Introduction

### Background

The Asian American community is currently one of the fastest growing populations in the nation [1]. They constitute a diverse population with multifaceted cultural, social, linguistic, familial, and religious values which span generations of acculturation [2]. Given this, many Asian American youth identify as third-culture children—straddling their American identity and that of their parents’ home country [3].

This unique positionality has both positive and negative implications for mental health. Overall, Asian Americans report lower rates of psychiatric disorders as compared to their white peers; however, the persistence of these conditions throughout one’s lifetime is similar between these two groups [4]. Serious mental illness, which is defined by the Substance Abuse and Mental Health Services Administration as a mental, emotional, or behavioral condition that significantly limits one’s functional ability and impedes one’s daily life, increased by almost two-fold among Asian American young adults (ages 18–25) between 2008 and 2018 [5]. Moreover, suicide was the most common cause of death among 15–24 year-old Asian American youth in 2019 [6]. In a study looking at the prevalence of psychiatric disorders among Asian American adults, 10.2% had a lifetime diagnosis of an anxiety or panic disorder, 9.5% had a lifetime diagnosis of a mood disorder, and 18.1% had a lifetime diagnosis of any mental health disorder [7].

Research on the disparities in Asian American mental health focuses on risk factors such as exposure to discrimination [8, 9], mental health stigma [10, 11], and poor rates of formal service utilization [12–14]. For Asian American youth, generational differences, language proficiency, ethnic marginalization, and family conflict have all been associated with increased depression [15, 16]. Meanwhile bicultural identity, personal spirituality, ethnic belonging, and enculturation have been shown to be protective against depression [16]. Furthermore, research has shown that peer relationships and ethnic identity may be protective against future depression and suicidal ideation among Filipino and Korean Americans [15]. Yet, there is little research that captures the richness of the Asian American community’s resiliency as a whole and how that might be elevated as a protective measure.

### Theoretical Framework

**Fig. 1:**
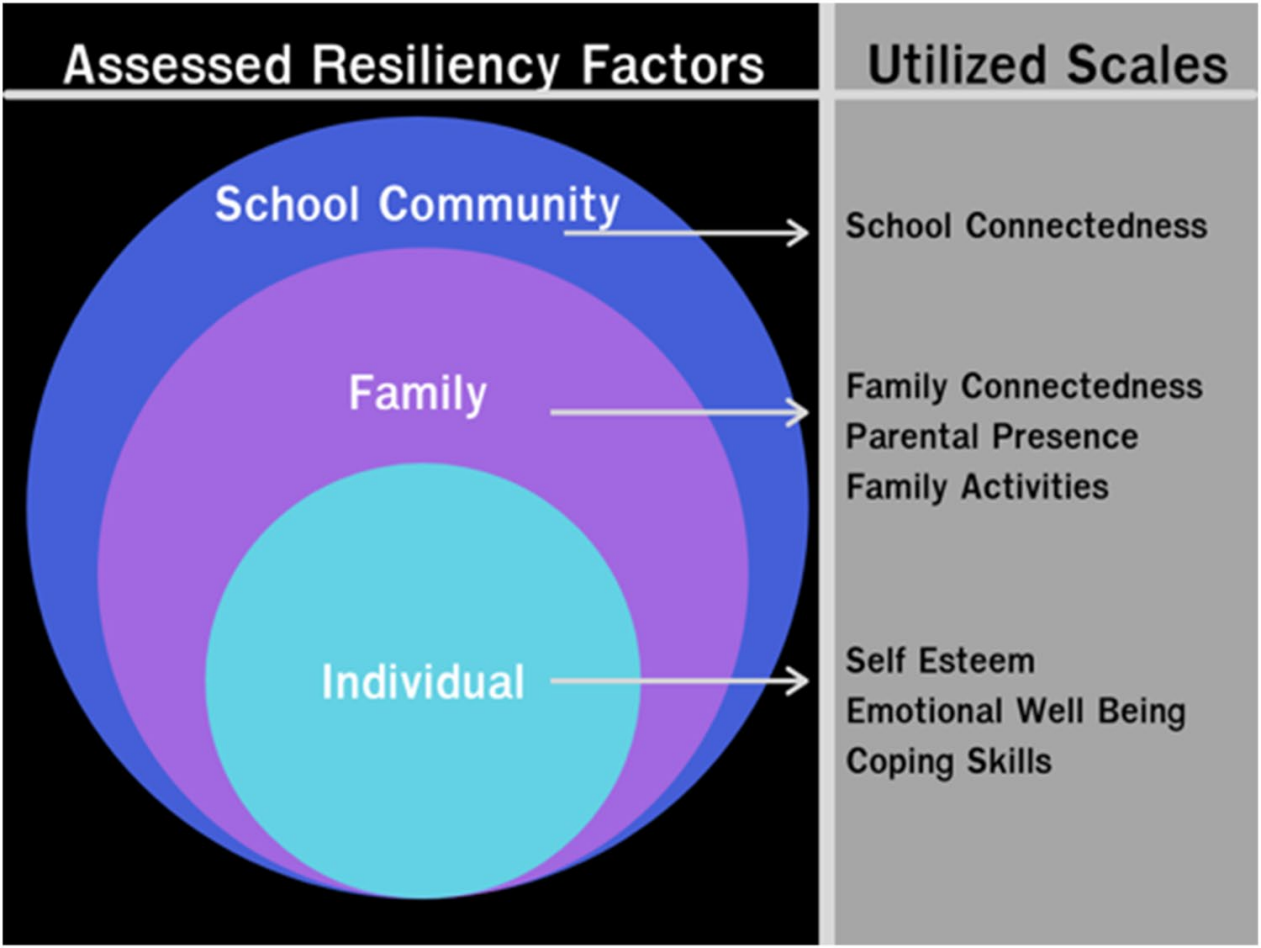
Conceptual framework of adolescent resiliency factors

Resiliency is the ability to adapt in the face of adverse experiences [17]. Resiliency can be built through protective factors that promote internal strength and connectedness [18]. A resiliency, rather than risk, framework centers the strengths and resources individuals, families, or communities employ to adapt to change and adversity [19, 20]. A socioecological framework provides a holistic and robust approach to exploring how to best support Asian Americans’ mental health throughout development (see Fig. 1) [21]. In a socioecological framework, protective factors can manifest on an individual (e.g., self-esteem, wellness), family (e.g., cohesion), and/or community (e.g., school inclusion) level in order to reduce behaviors that are harmful to the health and well-being of young people [17, 18, 17–18].

Previous literature has described resiliency as being fostered through protective factors that preserve one’s well-being through positive and productive engagement, connectedness with others, and optimized stress coping mechanisms [14].

While it has been repeatedly shown in the literature that Asian Americans underutilize mental health services and are at higher risk for adverse mental health symptomatology, there is limited research that provides insight on how to leverage this community’s assets in order to cultivate Asian American adolescents’ resiliency for future mental well-being in adulthood [24–26]. Furthermore, many existing studies on Asian American mental health utilize cross-sectional data and are not national, thus limiting the ability to understand associations between exposures and outcomes over time. Given these gaps in the literature, our study aims to determine the prospective associations between resiliency factors during adolescence within the context of the socioecological framework (individual, family, and community) and mental health outcomes, including depression, anxiety, and suicidality, in adulthood among a national sample of Asian Americans.

## Methods

### Participants

Our study utilized data from the National Longitudinal Study of Adolescent to Adult Health (Add Health) [27]. Add Health is a national, prospective cohort study that followed a U.S. population of adolescents into adulthood over five waves from 1994 to 2018. Data for the current study were from Wave I (1994–1995, adolescents aged 11–18 years) and Wave IV (2008–2009, adults aged 24–32 years).

The baseline (Wave I) Add Health study included 20,475 participants from 132 schools, recruiting from sites across the country. Among these participants, 1584 identified as Asian or Pacific Islander (Chinese, Korean, Filipino, Vietnamese, Indian, Japanese, & Other Asian) [28] and 1020 were followed through Wave IV. These 1020 participants who had data available for both Wave I and Wave IV comprised our final Asian American analytic sample.

The University of North Carolina School of Public Health Institutional Review Board (IRB) oversees the Add Health study. All participants provided informed consent in writing following IRB guidelines and the Code of Federal Regulations on the Protection of Human Subjects.

### Data Collection

As part of the Wave I data collection, students were asked to fill out an hour-long questionnaire within their classroom settings. This survey captured student responses about themselves, their family, and their community. Wave IV data was completed in 2008–2009 through in-home interviews with 15,701 study participants, aged 24–32. 92.5% of Wave IV participants were identified and located, of whom 80.3% were successfully interviewed. Higher rates of response were noted for US-born, white, and female participants. Add Health study investigators studied the difference between those who completed the interview and those who did not, and found that after study estimates were adjusted for final sampling weights, all study measures showed little relative and total bias for respondents vs. nonrespondents. The only area that showed significant differences was noted in those nonrespondents with low verbal capabilities, though this may be due to low total population in the Add Health sample. Add Health study investigators note that Wave IV sample bias is minimal, and that the population surveyed is comparable to that interviewed in Wave I [29]. These interviews included questions about mental health outcomes including depression, suicidality, and anxiety.

## Measures

### Sociodemographic Covariates

We considered sex, country of birth, age, household income, household size, sexual identity, and baseline depression/suicidality as co-variates. A detailed description of independent, dependent, measures and covariates can be found in the below, Table 1.

**Table 1:** Description of the measures

Demographic characteristics	
Age	Age in years was calculated from the date of birth and the date of the Wave I interview.
Sex	Assigned sex at birth was confirmed by interviewer at Wave I. Responses included (1) “male” (2) “female”. Two participants’ responses of “refused” and “don’t know” were excluded. Gender identity was not assessed separately from sex at baseline.
Sexual identity	Though sexual identity was measured at Wave I, it has been suggested that this data may suffer from “mischievous responses,” so Wave IV data on sexual identity was used as a more accurate report. [30] Sexual identity was classified based on an item in Wave IV asking participants to choose the description that best fit how participants identified. Response options included 100% heterosexual (straight), mostly heterosexual, bisexual, mostly homosexual, or 100% homosexual. We categorized responses as: (1) “heterosexual” for those that responded 100% heterosexual (straight) and (2) “sexual minority youth” for those that responded mostly heterosexual, bisexual, mostly homosexual, 100% homosexual [31].
Country of birth	Participants were asked “Were you born in the United States?” at Wave I. Responses included from (0) “yes” and (1) “no”. To maintain the sample size, those who refused, didn’t know, or skipped the question for legitimate reasons, meaning they already answered previously, were included in the (0) “yes” population.
Household income	Parents provided income information in the parental survey given in 1994 (same time as Wave I). There were asked “About how much total income, before taxes did your family receive in 1994? Include your own income, the income of everyone else in your household, and income from welfare benefits, dividends, and all other sources.”
Household size	Participants were asked to provide details of up to 20 household members at Wave I. The first of these questions for each member was “[#] Household Member: Is {NAME} male or female?” Responses included (0) “refused”, “legitimate skip”, “don’t know”,” not applicable” (1) “male” or (2)” female.” Participants who provided a response of (1) or (2) were considered as having that number of household members. If they responded to this question for multiple members, the total number of questions answered + 1 (for the participant themselves) was considered the final household size.
Baseline depression/suicidality	Composite of either depression score and/or suicidality: Depression Score was measured by a version of the Center for Disease Epidemiology-Depression scale (CES-D) in Wave I. The CES-D scale is a composite score of ten-items indicating the presence of depressive symptoms (e.g. “You felt that you could not shake off the blues, even with help from your family and your friend”). Four responses for each question ranged from (0) “never or rarely” to (3) “most of the time or all of the time,“ with higher scores indicating more depressive symptoms. A composite score of ≥ 10 indicated risk for clinical depression, so this was used as the cutoff [32]. The CES-D has been previously validated in adolescents and young adults[33]. During the past 12 months, have you ever seriously thought about committing suicide?” Responses included (0) “no” and (1) “yes.”
Asian race/ethnicity	Race/ethnicity was based on self-report. Those selecting Asian or Pacific Islander were asked to further describe their “Asian background” as Chinese, Vietnamese, Filipinx, Indian, Korean, Japanese, or Other. Participants could select multiple categories.

Primary predictor variables	
Individual factors	
Emotional well-being^a^	Scale created from the following questions (alpha 0.84–0.90) based on prior literature [34]: “How often was the following true during the past week? You were happy.” Responses ranged from (0) “never or rarely” to (3) “most of the time or all of the time.” “How often was the following true during the past week? You felt depressed.” Responses ranged from (0) “never or rarely” to (3) “most of the time or all of the time.” “How often was the following true during the past week? You felt hopeful about the future.” Responses ranged from (0) “never or rarely” to (3) “most of the time or all of the time.” “How often was the following true during the past week? You never get sad.” Responses ranged from (1) “strongly agree” to (5) “strongly disagree.” “How often was the following true during the past week? You felt lonely.” Responses ranged from (0) “never or rarely” to (3) “most of the time or all of the time.” Appropriate items were reverse-scored, and responses were summed to yield scale and score of 8 or more considered positive for emotional wellbeing [34]
Coping skills	Coping scale developed from avoidant (alpha 0.48), approach (alpha 0.21) and action (alpha 0.49) coping skills based on prior literature [35]. Reponses for all questions ranged from (1) “strongly agree” to (5) “strongly disagree.” Scale (5–25) created from the following questions: “You usually go out of your way to avoid having to deal with problems in your life.” “Difficult problems make you very upset.” “After carrying out a solution to a problem, you usually try to analyze what went right and when went wrong.” “When you get what you want, it’s usually because you worked hard for it.” “When making decisions, you usually go with your ‘gut feeling’ without thinking too much about the consequences of each alternative.” Appropriate items were reverse-scored, and responses were summed to yield a continuous variable (5–25), divided by five for ease of interpretation.
Self-esteem^a^	Modified Rosenberg Self-esteem Scale (RSES) (5–30) created from the following questions, based in prior literature [36, 41, 42]. Responses for all questions ranged from (1) “strongly agree” to (5) “strongly disagree”. Responses were summed to yield continuous variable, divided by five for ease of interpretation.[36, 41, 42] “You have a lot of good qualities.” “You have a lot to be proud of.” “You like yourself just the way you are.” “You feel like you are doing everything just about right.” “You feel socially accepted.” “You feel loved and wanted.”
*Family factors*	
Family connectedness	Family Parent Connectedness Scale (scores 5–50) created from the following ten questions (alpha 0.82) based in prior literature: [39, 40] Responses were summed to yield continuous variable, divided by five for ease of interpretation. “How much do you think she [resident mother] cares about you?“ Responses ranged from (1) “not at all” to (5) “very much” “How much do you think he [resident father] cares about you?“ Responses ranged from (1) “not at all” to (5) “very much” “Do you agree or disagree with the following statement? Overall, you are satisfied with your relationship with your mother.“ Responses ranged from (1) “strongly agree” to (5) “strongly disagree; reverse coded “Do you agree or disagree with the following statement? Overall, you are satisfied with your relationship with your father.“ Responses ranged from (1) “strongly agree” to (5) “strongly disagree; reverse coded “How close do you feel to your [mother]?” Responses ranged from (1) “not at all” to (5) “very much” “How close do you feel to your [father]?” Responses ranged from (1) “not at all” to (5) “very much” “How much do you feel that your parents care about you?” Responses ranged from (1) “not at all” to (5) “very much” “How much do you feel that people in your family understand you?” Responses ranged from (1) “not at all” to (5) “very much” “How much do you feel that you and your family have fun together?” Responses ranged from (1) “not at all” to (5) “very much” “How much do you feel that your family pays attention to you?” Responses ranged from (1) “not at all” to (5) “very much”
Parent-adolescent activities	Scale created from the following questions based in prior literature[34]. Responses for all questions ranged from (0) “no” to (1) “yes.” Responses were summed to yield continuous variable. [34] “Which of the following things have you done with your [mother or father]: have you gone shopping?” “Which of the following things have you done with your [mother or father]: have you played a sport?” “Which of the following things have you done with your [mother or father]: have you gone to a religious service or church-related event?” “Which of the following things have you done with your [mother or father]: have you gone to a movie, play, museum, concert or sports event?”
Parental presence	Scale created from the following questions based in prior literature. Responses for all questions ranged from (1) “always” to (5) “never.” Responses were summed to yield continuous variable.[34] “How often is [mother or father] at home when you leave for school.” “How often is [mother or father] at home when you return from school.” “How often is [mother or father] at home when you go to bed.”
*Community factors*	
School connectedness	School Connectedness Scale (scores 5–25) created from the five following questions (alpha 0.78)[38] based in prior literature. Responses ranged from (1) “strongly agree” to (5) “strongly disagree” for all questions. Responses were summed to yield continuous variable, divided by five for ease of interpretation: “The teachers at your school treat students fairly. Last year, the teachers at your school treated students fairly.“ “You feel like you are part of your school. Last year, you felt like you were a part of your school.” “You feel close to people at your school. Last year, you felt close to people at your school.” “You are happy to be at your school. Last year, you were happy to be at your school.” “You feel safe in your school. Last year, you felt safe in your school”
*Primary outcome variables*	
Depression symptoms^a^	Depression Score was measured by a version of the Center for Disease Epidemiology-Depression scale (CES-D) in Wave IV. The CES-D scale is a composite score of ten-items indicating the presence of depressive symptoms (e.g. “You felt that you could not shake off the blues, even with help from your family and your friends”). Four responses for each question ranged from (0) “never or rarely” to (3) “most of the time or all of the time,“ with higher scores indicating more depressive symptoms. A composite score of ≥ 10 indicated risk for clinical depression, so this was used as the cutoff. [32] The CES-D has been previously validated in adolescents and young adults [33].
Depression diagnosis^a^	“Has a doctor, nurse, or other health care provider ever told you that you have or had: depression?” Responses ranged included (0) “no” and (1) “yes.”
Anxiety or panic diagnosis^a^	“Has a doctor, nurse, or other health care provider ever told you that you have or had: anxiety or panic disorder?” Responses ranged included (0) “no” and (1) “yes.”
Suicidality^a^	“During the past 12 months, have you ever seriously thought about committing suicide?” Responses ranged included (0) “no” and (1) “yes.”
Mental health outcome	A report of either depressive symptoms (CES-D score of 10 or greater), depression diagnosis, anxiety or panic diagnosis, or suicidality was considered a mental health outcome.

^a^For the calculation of percentages and adjusted odds ratios, the responses were dichotomized based on a clinical cutoff or natural cutoffs with the responses or data

### Primary Exposure Variable: Resiliency

Our study’s primary independent variable was based on the socioecological model of resiliency–the ability to positively adapt even through the experience of hardship [17]. Building upon previous literature, the protective factors chosen were mapped to the validated questionnaire and responses from the Add Health codebook [23, 34–36]. Using prior research looking at Add Health data and adolescent resiliency as a guide, protective factors were grouped into individual, family, and school-community level measures [37]. The measures comprising our primary predictor variable of resiliency factors include the school connectedness scale [38], family connectedness scale [39, 40], parental presence scale [34], family activities scale [34], self-esteem scale [36, 41, 42], emotional well-being scale [34], and coping skills scale [35], all of which have been previously described in the literature.

### Primary Outcome Variable: Mental Health Outcomes

Our study’s primary outcome variable of adult mental health outcomes comes from self-reported at-home interviews conducted during Wave IV with participants aged 24–32 in 2008–2009. The “mental health outcomes” variable includes any of the following: self-reported lifetime diagnosis of depression, lifetime diagnosis of anxiety or panic disorder, suicidality, and/or having a positive Center for Epidemiologic Studies Depression scale (CES-D-10) for current depressive symptoms [23, 43].

### Statistical Analysis

Descriptive statistics were performed to characterize the sample. Unadjusted associations between each resiliency factor (Wave I), as the independent variable, and mental health outcomes (Wave IV), as the dependent variable, were determined using multiple logistic regression analyses. Resiliency factors associated with mental health outcomes at Wave IV were examined in adjusted logistic regression analyses. Adjusted analyses controlled for sociodemographic variables that were associated with mental health outcomes (Wave IV) in univariate analyses, including sexual minority status and baseline depression or suicidality (participants were not asked at baseline about anxiety or depressive symptoms). When checking for statistical assumptions, we were not able to include emotional well-being, self-esteem, and family connectedness in the same model because they were highly multicollinear (variance inflation factors > 50); thus, separate models were run for these predictors. Two-sided alpha was set at 0.05. All analyses utilized nationally representative sample weighting and were conducted using Stata 15.1.

## Results

Of the 1020 study participants with Wave I and Wave IV data who self-identified as Asian American or Pacific Islander, 20.4% identified as Chinese, 38.7% Filipino, 9.4% Japanese, 4.2% Asian Indian, 8.9% Korean, 6.9% Vietnamese, and 25.5% other Asian. The mean age ± standard error of participants at baseline was 15.6 ± 0.25 years. Just over half the participants were male (52%) and about 11% identified as sexual minorities in adulthood. Mean household size was 5.0 ± 0.26 people and average household income was 49.7 ± 2.39 thousands of dollars at baseline. Almost 60% of participants were born in the United States. The mean score of resiliency factor scales at baseline (Wave I) are grouped using the socioecological framework: individual (emotional wellbeing = 0.77 ± 0.02, coping skills = 3.20 ± 0.03, self-esteem = 4.78 ± 0.04), family (family connectedness = 8.44 ± 0.06, parental presence = 2.26 ± 0.05, family activities = 1.62 ± 0.07), and school community (school connectedness = 3.75 ± 0.04) levels. At Wave I, almost half of the participants reported depression or suicidality as opposed to Wave IV where about one-third of the respondents experienced mental health outcomes (Table 2).

**Table 2:** Baseline demographic characteristics, exposures, and outcomes at fourteen-year follow-up among Asian American participants in the National Longitudinal Study of Adolescent to Adult Health

N	1020
*Demographic variables (Wave I)*	Mean ± SE / %
Age (years)	15.6 ± 0.25
Household income (thousands of dollars)	49.7 ± 2.39
Household size (number of people)	5.0 ± 0.26
Country of birth	
United States	59.9%
Other	40.1%
Asian background	
Chinese	20.4%
Filipino	38.7%
Japanese	9.4%
Asian Indian	4.2%
Korean	8.9%
Vietnamese	6.9%
Other Asian	25.5%
Assigned Sex at Birth	
Female	48.0%
Male	52.0%
Sexual Identity* (at Wave IV)	
Sexual minority	11.3%
Heterosexual	88.7%
Baseline depression and/or suicidality	48.4%
*Exposure variables (Wave I)*	
Individual level resiliency factors	
Emotional well-being	0.77 ± 0.02
Coping skills	3.20 ± 0.03
Self-Esteem	4.78 ± 0.04
Family level resiliency factors	
Family connectedness	8.44 ± 0.06
Parental presence	2.26 ± 0.05
Family activities	1.62 ± 0.07
School community level resiliency factors	
School connectedness	3.75 ± 0.04
*Outcome variable (Wave IV)*	
Mental health outcome	33.4%

All means and percentages were calculated with weighted data to reflect the representative proportion in the target U.S. population.

*Though sexual identity was measured at Wave 1, it has been suggested that this data may suffer from “mischievous responses,” so Wave IV data on sexual identity was used as a more accurate report. [30] Sexual minority includes those who identify as gay, bisexual, and mostly heterosexual

In logistic regression analyses (Table 3), higher self-esteem in adolescence was associated with more than two-fold lower odds of poorer mental health outcomes in adulthood amongst study participants in both unadjusted and adjusted models (odds ratio [OR] 0.44, 95% confidence interval [CI] = 0.31–0.61; adjusted odds ratio [AOR] 0.54, 95% CI = 0.37–0.79). Similarly, greater levels of family connectedness during adolescence were associated with reduced odds of poor mental health outcomes in adulthood in both adjusted and unadjusted models (OR 0.69, 95% CI = 0.59–0.81; AOR 0.78, 95% CI = 0.65–0.93). Higher levels of adolescent emotional well-being were also associated with 60% lower odds of poorer adult mental health outcomes; however, this association was diminished and no longer significant after adjusting for potential confounders (OR 0.42, 95% CI = 0.26–0.69; AOR 0.66, 95% CI = 0.36–1.19). Significant associations were not found between coping skills, parental activities, school connectedness, or parent presence in adolescence and adult mental health outcomes.

**Table 3:** Protective resiliency factors in adolescence associated with mental health outcomes in young adulthood of Asian American participants in the National Longitudinal Study of Adolescent to Adult Health

	Mental Health Outcomes (i.e., self-reported diagnosis of depression, anxiety or panic disorder, suicidality, or current symptoms of depression)			
	Unadjusted		Adjusted*	
	OR (95% CI)	p value	AOR (95% CI)	p value
Covariates (reference / non-reference)				
Age	0.93 (0.83–1.05)	0.242		
Sex (female / max)	1.59 (0.95–2.66)	0.075		
Parent Education (college or more / high school or less)	1.29 (0.75–2.23)	0.349		
Household Income	1.00 (0.99-1.00)	0.316		
Country of Birth (U.S. / not U.S. )	0.72 (0.45–1.16)	0.177		
Household Size	1.00 (0.92–1.10)	0.915		
Sexual Identity (heterosexual / sexual minority)	**0.33 (0.18–0.62)**	**0.001**		
Baseline depression & suicidality	**2.79 (1.89–4.11)**	**< 0.001**		
Individual predictors				
Emotional well-being scale	**0.42 (0.26–0.69)**	**0.001**	0.66 (0.36–1.19)	0.165
Coping skills scale	0.73 (0.48–1.13)	0.160		
Self-esteem scale	**0.44 (0.31–0.61)**	**< 0.001**	**0.54 (0.37–0.79)**	**0.002**
Family predictors				
Family connectedness scale	**0.69 (0.59–0.81)**	**< 0.001**	**0.78 (0.65–0.93)**	**0.005**
Parental presence scale (before school/after school bedtime)	0.86 (0.66–1.12)	0.261		
Family activities scale (0–4 activities/month)	0.88 (0.71–1.08)	0.218		
School community predictors				
School connectedness scale	0.87 (0.65–1.17)	0.352		

Statistically significant values are given in bold

Data are from the National Longitudinal Study of Adolescent to Adult Health (Add Health) Waves I and IV

*OR* odds ratio,* AOR* adjusted odds ratio,* CI* confidence interval

*Multiple logistic regression analyses were used to identify associations between mental health outcomes at 14-year follow-up and baseline adolescent resiliency factors, adjusting for sexual identity and baseline depression/suicidality. Separate adjusted models were run for emotional well-being, self-esteem, and family connectedness due to multicollinearity

## Discussion

Our study shows that several individual and family adolescent resiliency factors present during adolescence are strong predictors of better mental health outcomes in early adulthood amongst the Asian American community. Particularly, self-esteem (individual) and family connectedness (family) were protective against mental health outcomes independent of other demographic and clinical factors. Among Asian American adolescents, self-esteem was associated with a roughly 50% lower odds of mental health outcomes in early adulthood. Similarly, family connectedness in adolescence was linked to four-fold lower odds of mental health outcomes.

Despite the known mental health burden among the Asian American population, many Asian Americans with mental health needs continue to have the lowest rates of formal treatment [13, 44], with evidence suggesting that Asian American immigrants are more likely to rely on informal supports or services [8]. Since mental health services have been chronically underutilized by the Asian American community [19, 20], protective resiliency factors like family connectedness and self-esteem should be cultivated among Asian American adolescents to promote positive psychological adaptation.

These findings are complemented by prior research and theory showing the importance of individual, family, and community level resiliency factors in preventing poor mental health outcomes within a socioecological model. Our results are consistent with established literature demonstrating that higher self-esteem, an individual level resiliency factor, is associated with better mental health [45–47]. It is important to note that prior literature has indicated that despite reporting the highest levels of personal and parental education, Asian American adolescents commonly report the lowest self-esteem scores compared to all other racial/ethnic subgroups [48]. Moreover, it has been previously demonstrated that self-esteem mediates the relationship between discrimination and depression, specifically in Asian American second generation immigrants [46]. One study looking at Chinese-American young adults shows that strong cultural identity, through multilingualism and pride in heritage, is positively associated with higher self-esteem [49]. With the context of these prior findings, our results indicated that improving mental health among the Asian American community requires dismantling the myth of the “Model Minority” and developing positive adolescent self-esteem through culturally relevant means in a way that strengthens resiliency at an individual level [50].

Research has suggested that strong family relationships have been protective against suicide attempts by Asian American adolescents, depending on the level of acculturation [51]. However, in a study addressing the unmet mental health needs of urban Asian American adolescents, family support was identified by service providers as the most prevalent need [52]. Our results amplify this identified gap in holistic care, suggesting that providing scaffolding to bolster family connectedness can be suppressive of future mental health disease.

This research has several strengths including its large and diverse participant sample, use of validated measures, asset-based approach, and longitudinal design. However, several limitations should be addressed. Since this was prospective cohort study, the associations between resiliency factors and mental health outcomes in the Asian American community may be affected by confounding variables, and causality cannot be established. Additionally, while we controlled for baseline depression/suicidality, we could not factor in lifetime diagnosis of depression or anxiety as we did in our primary outcome variable due to the fact these components were not collected in Wave I. However, we controlled for several potential sociodemographic confounders in the regression models. Given the vast diversity of the Asian American population, another limitation of our study is that our results are not disaggregated by any of the over 20 origin groups that constitute this community [2] due to the small sample sizes and insufficient power in each of the possible subgroups. The breakdown of Asian subgroups in our data does not fully capture the demographics of this population in the United States [2]; however, survey weighting was implemented as a means by which to mitigate the lack of representative data. Additionally, the socioecological model of resiliency may include additional individual, family, and community factors that might also be important in predicting adult mental health outcomes, which were not collected in Add Health [26].

## New Contributions to the Literature

By utilizing a large, nationally representative study population and a 14-year, longitudinal, prospective analysis, this study provides insight on how to best identify Asian American adolescents’ strengths in order to support their adult mental health outcomes. Our results provide new insight on the importance of adolescent self-esteem, an individual level resiliency factor, and family connectedness, a family level resiliency factor, in protecting against adverse mental health outcomes in the Asian American population. Applying the socioecological model of resilience with Asian Americans is a novel approach to framing the factors that clinicians, educators, and caregivers should consider when addressing behavioral, emotional, or psychological issues within this population.

The Asian American community has been historically underrepresented and understudied in scientific research [53, 54]. Given the growing reports of discrimination against Asian Americans and hate crimes directed towards this population during the current COVID-19 pandemic [55–57], identifying ways to foster this community’s resilience and mental health is crucial. Our results may have important implications for clinical practice and mental health policy. Culturally relevant ways by which trusted adults can help foster self-esteem among Asian American youth and by which anchor institutions can provide guidance for primary caretakers in building family connection, should be considered as possible interventions. Leveraging the socioecological model of resilience with Asian American adolescents may be a way to prevent negative mental health outcomes in adulthood. Helping develop skills for both caretakers and their youth can promote resilience on a family and individual level. That being said, further research is still needed to explore the relationship between resiliency factors in Asian American youth stratifying by subgroup to study the impact of these communities’ unique cultural, linguistic, and social identities on adult mental health outcomes.

## Data Availability

This study analyses restricted-use data from Add Health. Persons interested in obtaining Data Files from Add Health should contact Add Health, The University of North Carolina at Chapel Hill, Carolina Population Center, 206 W. Franklin Street, Chapel Hill, NC 27,516 − 2524 (addhealth_contracts@unc.edu). Further information on how to obtain the Add Health data files is available on the Add Health website (http://www.cpc.unc.edu/addhealth). The authors did not receive special access privileges to the data that others would not have.
